# Assessment of a Staging System for Sigmoid Colon Cancer Based on Tumor Deposits and Extramural Venous Invasion on Computed Tomography

**DOI:** 10.1001/jamanetworkopen.2019.16987

**Published:** 2019-12-06

**Authors:** Nigel D’Souza, Annabel Shaw, Amy Lord, Svetlana Balyasnikova, Muti Abulafi, Paris Tekkis, Gina Brown

**Affiliations:** 1 Department of Colorectal Surgery, Croydon University Hospital, London, United Kingdom; 2 Imperial College, London, United Kingdom; 3 Department of Gastrointestinal Imaging, Royal Marsden Hospital, London, United Kingdom; 4 Department of Colorectal Surgery, Royal Marsden Hospital, London, United Kingdom

## Abstract

**Importance:**

Preoperative TNM stratification of colon cancer on computed tomography (CT) does not identify patients who are at high risk of recurrence that could be selected for preoperative treatment.

**Objective:**

To evaluate the utility of CT findings for prognosis of sigmoid colon cancer.

**Design, Setting, and Participants:**

This prognostic study used retrospective data from patients who underwent bowel resection for sigmoid colon cancer between January 1, 2006, and January 1, 2015, at a tertiary care center receiving international and national referrals for colorectal cancer. Statistical analysis was performed in April 2019.

**Main Outcomes and Measures:**

Cox proportional hazards regression analysis was performed to investigate CT findings associated with disease recurrence. Kaplan-Meier survival plots were calculated for disease-free survival using CT staging systems.

**Results:**

Of the 414 patients who had sigmoid colon cancer (248 [60.0%] men; mean [SD] age, 66.1 [12.7] years), with median follow-up of 61 months (interquartile range, 40-87 months), 122 patients (29.5%) developed disease recurrence. On multivariate analysis, nodal disease was not associated with disease recurrence; only tumor deposits (hazard ratio [HR], 1.90; 95% CI, 1.21-2.98; *P* = .006) and extramural venous invasion (HR, 1.97; 95% CI, 1.26-3.06; *P* = .003) on CT were associated with disease recurrence. Significant differences in disease-free survival were found using CT-T3 substage classification (HR, 1.88; 95% CI, 1.32-2.68) but not CT-TNM (HR, 1.55; 95% CI, 0.94-2.55). The presence of tumor deposits or extramural venous invasion on CT (HR, 2.45; 95% CI, 1.68-3.56) had the strongest association with poor outcome.

**Conclusions and Relevance:**

In this study, T3 substaging and detection of tumor deposits or extramural venous invasion on preoperative CT scans of sigmoid colon cancer were prognostic factors for disease-free survival, whereas TNM and nodal staging on CT had no prognostic value. T3 substaging and detection of tumor deposits or extramural venous invasion of sigmoid colon cancer was superior to TNM on CT and could be used to preoperatively identify patients at high risk of recurrence.

## Key Points

**Question:**

Can prognostic features on preoperative computed tomography (CT) identify high-risk sigmoid colon cancer?

**Findings:**

In this prognostic study of 414 patients with nonmetastatic disease, standard CT TNM assessment was not prognostic. Differences in disease-free survival were found using T3 substaging on CT, but most significantly by evidence of tumor deposits and extramural venous invasion on CT.

**Meaning:**

The results suggest that T3 substaging and identification of tumor deposits and extramural venous invasion on CT may be prognostic factors and that patients with these findings may benefit from preoperative treatment of sigmoid colon cancer.

## Teaser

This prognostic study assesses whether preoperative computed tomographic findings can be used for sigmoid colon cancer prognosis.

## Introduction

There is increasing awareness that preoperative identification of high-risk tumors on imaging has improved rectal cancer outcomes.^1,2,3^ To date, such a strategy does not exist for colon cancer and may partly explain why rectal cancer outcomes have improved and now have overtaken colon cancer outcomes.^4,5,6,7^ Novel treatment options for advanced, nonmetastatic colon cancer, such as neoadjuvant chemotherapy,^8^ complete mesocolic excision, or hyperthermic intraperitoneal therapy,^9^ may improve outcomes among patients with an otherwise poor prognosis but require accurate preoperative imaging to identify locally advanced tumors. At present, preoperative clinical staging of colon cancer by computed tomography (CT) is based on the TNM system.^10^

Nodal disease, which is the traditional histopathologic indication for adjuvant chemotherapy, is no longer recommended for clinical staging^11^ because it cannot be reliably identified by CT, with specificity of 55% to 67% on meta-analysis.^12,13,14^ More recently, DNA analysis of primary tumor, lymph nodes, and metastases in patients with colorectal cancer has found that most distant metastases had an origin different from lymph nodes.^15^

Tumor stage has traditionally not been seen to be relevant because it was not prognostic. In 2007, advanced T3 (>5-mm depth of spread beyond the muscularis propria) and T4 categories were identified as criteria for colon cancer with poor prognosis on CT.^16^ Patients with advanced T3 and T4 tumors identified on CT had worse recurrence-free survival at 3 years than patients with T1 and T2 tumors (53%) and those with early T3 tumors (87%). The use of preoperative treatment for all patients with T3 tumors was recommended in a recent colon cancer trial,^8,17^ but the preoperative treatment failed to reduce recurrence; this finding may have been associated with inclusion of a group at low risk of recurrence (early T3 tumors with <5-mm spread).

With the emergence of novel and varied treatment strategies, an accurate preoperative staging system permits an opportunity to stratify treatment as well as surveillance. Risk factors for recurrence that were not evaluated by TNM are now standardly reported on CT report proformas for colon cancer,^18^ including advanced T3 substage,^16,19^ localized peritoneal infiltration,^20,21^ extramural venous invasion (EMVI),^19,22,23^ and discontinuous tumor deposits.^24,25^ In this study, we aimed to investigate whether these known disease features on CT are associated with oncologic outcome of sigmoid colon cancer and whether they could be used to optimize preoperative staging systems.

## Methods

### Data Sources and Baseline Variables

This prognostic study used retrospective data from patients identified in a prospectively maintained institutional database at the Royal Marsden Hospital, London, England, that included patient data from national and international tertiary referral centers in addition to a local referral network of 6 hospitals within Greater London: Epsom and St Heliers University Hospitals, Croydon University Hospital, Kingston Hospital, St George’s Hospital, and Chelsea and Westminster Hospital. Local ethical approval was obtained from the Royal Marsden Hospital Committee for Clinical Research. The need for informed consent was waived by the committee because this was a retrospective study of clinical practice. This study followed the Reporting Recommendations for Tumor Marker Prognostic Studies (REMARK).^26^

All patient information was discussed during a central colorectal cancer multidisciplinary team meeting. Treatment plans were individualized after multidisciplinary team discussion, except for the operative strategy if the patients had already undergone a surgical procedure as an emergency or were referred specifically for adjuvant treatment. The study start date was January 1, 2006, when modern, high-resolution, multidetector CT scanners became commonplace. The study end date was January 1, 2015; thus, patients had at least 3 years of follow-up data commencing from the date of their surgical procedure. Standardized follow-up for English National Health Service patients was performed according to the National Institute for Health and Care Excellence guidelines for colorectal cancer.^27^

Patients were eligible for inclusion if they had nonmetastatic adenocarcinoma arising in the sigmoid colon on CT scan and underwent colonic resection. The mesorectal-mesocolic junction as recognized by the sigmoid take-off^28^ was used as the distal landmark to define sigmoid tumors. Exclusion criteria included synchronous tumors, metastatic disease, early tumors removed with endoscopic treatment only, or disease that was later found not to be colorectal cancer. Patients who received palliative treatment without resection of their primary tumor and patients who died within 30 days of undergoing a surgical procedure were also excluded. If no follow-up data were available for international or national patients who returned to their referral hospital after undergoing a surgical procedure, they were excluded. Data were extracted on basic demographic characteristics, such as age, sex, emergency presentation, and preoperative or postoperative treatment (ie, radiotherapy or chemotherapy).

### Radiologic Analysis

Computed tomography was performed at National Health Service and international institutions on multidetector CT scanners from a range of manufacturers using standard CT abdomen-pelvis protocols with 1-mm section intervals in more than 90% of cases. Images were viewed with multiplanar reconstruction when possible; this was a limitation in fewer than 10% of cases. Data on CT reports from the local hospital were extracted when reported by gastrointestinal radiologists on standardized CT reporting proformas for colon cancer developed by the Royal College of Radiologists.^18^ Results were otherwise rereported by 2 gastrointestinal radiologists who had more than 5 years of experience and who were blinded to the clinical information. The CT data were recorded on T stage (including T3 substaging by depth of spread), nodal disease, discontinuous tumor deposits, EMVI, and peritoneal infiltration localized to the tumor site (ie, not disseminated). The T3 substage was classified according to the TNM guidelines for colorectal cancer,^10^ measuring extramural tumor spread in millimeters beyond the muscularis propria: T3a (<1-mm spread), T3b (1- to 5-mm spread), T3c (>5- to 15-mm spread), and T3d (>15-mm spread).

Two CT-staging classifications for poor and good prognosis groups based on T stage have previously been described.^8,16,19,20,29^ The CT-TNM system uses conventional TNM staging and classifies all T3 and T4 tumors to a poor prognosis group and all T1 and T2 tumors to a good prognosis group. The CT-TNM system was used to select patients with T3 or T4 colon cancer for preoperative therapy by a prospective multicenter trial.^8^ The CT-T3 substage system combines high-risk T3 substage tumors (T3c-T3d) and T4 tumors to create a poor prognosis group, whereas good prognosis T3 substage tumors (T3a-T3b) are grouped with T1 and T2 tumors. The CT-T3 substage system has been described in previous reports,^16,19,20,29^ with high sensitivity and specificity for accurate substaging on meta-analysis.^12^

### Pathologic Analysis and Clinical End Points

Pathologic variables of T stage, nodal disease, tumor deposits, differentiation grade, EMVI, peritoneal disease, and circumferential resection margin status were recorded on the standard data set issued by the Royal College of Pathologists and extracted.^30^ Data were extracted on disease recurrence and death from clinical medical notes. Recurrence site was confirmed on imaging and was reclassified as local recurrence or distant recurrence.

### Statistical Analysis

The primary end point was to investigate the association between known preoperative prognostic factors on CT and disease recurrence: local recurrence, distant recurrence, and overall (local or distant recurrence). The secondary end point was to compare the prognostic accuracy of different CT staging systems based on TNM, T3 substage, and a CT staging system based on significant risk factors for disease-free survival on multivariate analysis. All included patients had complete data for radiologic staging. Local institutions were contacted to seek missing data particularly on patient follow-up. Missing data for pathologic variables were quantified. Patients who were lost to follow-up had the duration of follow-up quantified as the period between the date of surgical procedure and date of the last clinic appointment or radiologic scan. Survival analysis was defined for disease-free survival as time to event in months from the date of surgical procedure to the date of local recurrence, distant recurrence, or death from colorectal cancer. Observations were censored at the date of last follow-up or the date of death from noncolorectal cancer. For local or distant recurrence, time to recurrence was measured from the date of surgical procedure to the date of local or distant recurrence; patients with no disease recurrence were censored at the date of last follow-up or death.

Statistical analysis was performed using Stata, version 13 (StataCorp LLC). Frequency tables were compiled of all prognostic variables and outcomes. Continuous variables were compared using a 2-tailed *t* test. Median survival was compared using the Wilcoxon rank sum test. Categorical variables were compared using Fisher exact test. Statistical significance was set as *P* < .05 in 2-tailed tests. Clinically relevant covariates were chosen a priori to test higher-order interaction. Age was recategorized (<55 years, 55 to <70 years, 70-85 years, and >85 years) based on clinically relevant infection points for survival. Model building used backward stepwise selection (*P* < .20) with sequential elimination of nonsignificant predictor variables (*P* > .05) and likelihood ratios to test variable inclusion or exclusion decisions. Kaplan-Meier survival plots were calculated for recurrence-free survival. Cox multiregression analysis was used to derive univariate and multivariate hazard ratios (HRs) for recurrence from preoperative variables.

## Results

Between January 1, 2006, and January 1, 2015, information on 780 patients with suspected sigmoid colon cancer on radiologic imaging was discussed in the multidisciplinary team meeting, of whom 414 patients (248 [60.0%] men; mean [SD], age, 66.1 [12.7] years) met the inclusion criteria (eFigure in the Supplement) and had complete data (Table 1). Median follow-up was 61 months (interquartile range, 40-87 months).

**Table 1.** Frequency of Prognostic Findings and Outcomes

Variable and Outcome	Type of Computed Tomography, No. (%)			
	TNM (N = 414)		T3 Substage (N = 414)^a^	
	Good Prognosis Group (n = 86)	Poor Prognosis Group (n = 328)	Good Prognosis Group (n = 259)	Poor Prognosis Group (n = 155)
Age, mean (SD), y	65.6 (11.6)	66.2 (13.0)	66.2 (12.2)	65.8 (13.5)
Male	49 (57)	199 (61)	158 (61)	90 (58)
Emergency presentation	1 (1)	48 (15)	20 (8)	29 (19)
Neoadjuvant therapy	2 (2)	40 (12)	12 (5)	30 (19)
Computed tomography finding				
T stage				
1	20 (23)	0	20 (8)	0
2	66 (77)	0	66 (25)	0
3	0	255 (78)	173 (67)	82 (53)
4	0	73 (22)	NA	73 (47)
N stage				
0	76 (88)	144 (44)	182 (70)	38 (25)
1	6 (7)	86 (26)	54 (21)	38 (25)
2	1 (1)	20 (6)	4 (2)	17 (11)
1c, tumor deposits	3 (4)	78 (24)	19 (7)	62 (40)
EMVI	6 (7)	164 (50)	42 (16)	128 (83)
Localized peritoneal disease	0	31 (9)	0	31 (20)
pT				
1	19 (22)	5 (2)	23 (9)	1 (1)
2	33 (38)	29 (9)	58 (22)	4 (3)
3	30 (35)	202 (61)	148 (57)	84 (54)
4	4 (5)	92 (28)	30 (12)	66 (43)
pN				
0	58 (67)	193 (59)	169 (65)	82 (53)
1	24 (28)	77 (23)	61 (24)	40 (26)
2	4 (5)	49 (15)	25 (10)	28 (18)
1c, tumor deposits	0	9 (3)	4 (2)	5 (3)
pEMVI present	15 (17)	127 (39)	60 (23)	82 (53)
Grade				
Well or moderate	75 (87)	294 (90)	232 (90)	137 (88)
Poor	5 (6)	25 (8)	14 (5)	16 (10)
No residual	5 (6)	3 (1)	7 (3)	1 (1)
Missing or other	1 (1)	6 (2)	6 (2)	1 (1)
R1 or R2	0	10 (3)	3 (1)	7 (5)
Leak	3 (3)	11 (3)	9 (4)	5 (3)
Adjuvant	24 (28)	180 (55)	108 (42)	94 (61)
Local recurrence	8 (9)	44 (13)	22 (8)	30 (19)
Distant recurrence	13 (15)	88 (27)	50 (19)	51 (33)

Abbreviations: EMVI, extramural venous invasion; NA, not applicable; p, pathologic (mode of determining criteria for tumor description); R1, microscopic residual tumor; R2, macroscopic residual tumor.

^a^ T3 defined as greater than 5-mm depth of spread beyond the muscularis propria.

On univariate analysis, the preoperative CT findings associated with overall recurrence included T stage (HR, 1.24; 95% CI, 1.12-1.39; *P* < .001), N stage (HR, 1.51; 95% CI, 1.27-1.80; *P* < .001), EMVI (HR, 2.69; 95% CI, 1.87-3.86; *P* < .001), tumor deposits (HR, 2.95; 95% CI, 2.04-4.29; *P* < .001), and peritoneal disease (HR, 2.22; 95% CI, 1.31-3.76; *P* = .003) (Table 2). Computed tomography findings of T stage (HR, 1.18; 95% CI, 1.05-1.32; *P* < .001), N stage (HR, 1.48; 95% CI, 1.13-1.92; *P* = .004), EMVI (HR, 2.75; 95% CI, 1.56-4.83; *P* < .001), tumor deposits (HR, 3.24; 95% CI, 1.86-5.64; *P* < .001), and peritoneal disease (HR, 4.37; 95% CI, 2.29-8.33; *P* < .001) were significantly associated with local recurrence (Table 2). Emergency presentation (HR, 1.43; 95% CI, 1.10-1.86; *P* = .007) as well as the preoperative CT findings of T stage (HR, 1.14; 95% CI, 1.05-1.24; *P* < .001), N stage (HR, 1.55; 95% CI, 1.28-1.87; *P* < .001), EMVI (HR, 2.89; 95% CI, 1.94-4.36; *P* < .001), and tumor deposits (HR, 2.90; 95% CI, 1.94-4.36; *P* < .001) (Table 2) were significantly associated with distant recurrence.

**Table 2.** Univariate and Multivariate Analysis of Preoperative Variables for Disease Recurrence

Preoperative Variable	Type of Recurrence					
	Overall		Local		Distant	
	Hazard Ratio (95% CI)	*P* Value	Hazard Ratio (95% CI)	*P* Value	Hazard Ratio (95% CI)	*P* Value
**Univariate Analysis**						
Age	1.00 (0.99-1.00)	.98	0.99 (0.99-1.01)	.90	1.00 (0.99-1.00)	.96
Sex	0.84 (0.58-1.21)	.34	1.10 (0.63-1.91)	.74	0.73 (0.48-1.10)	.14
Neoadjuvant therapy	1.34 (0.79-2.27)	.27	0.90 (0.36-2.26)	.83	1.34 (0.75-2.39)	.33
Emergency presentation	1.44 (1.13-1.83)	.003	1.44 (0.99-2.06)	.05	1.43 (1.10-1.86)	.007
Computed tomography finding						
T stage	1.24 (1.12-1.39)	<.001	1.18 (1.05-1.32)	<.001	1.14 (1.05-1.24)	<.001
N stage	1.51 (1.27-1.80)	<.001	1.48 (1.13-1.92)	.004	1.55 (1.28-1.87)	<.001
Extramural venous invasion	2.69 (1.87-3.86)	<.001	2.75 (1.56-4.83)	<.001	2.89 (1.94-4.34)	<.001
Tumor deposits	2.95 (2.04-4.29)	<.001	3.24 (1.86-5.64)	<.001	2.90 (1.94-4.36)	<.001
Peritoneal disease	2.22 (1.31-3.76)	.003	4.37 (2.29-8.33)	<.001	1.32 (0.67-2.62)	.42
TNM stage	1.57 (0.96-2.60)	.08	1.42 (0.67-3.02)	.36	1.83 (1.02-3.28)	.04
T3 substage^a^	1.88 (1.32-2.68)	<.001	2.43 (1.40-4.22)	.002	1.93 (1.31-2.86)	.001
TDV stage	2.81 (1.95-4.05)	<.001	2.67 (1.52-4.70)	.001	3.08 (2.05-4.64)	<.001
**Multivariate Analysis**						
Computed tomography finding						
Tumor deposits	1.90 (1.21-2.98)	.006	2.84 (1.62-5.01)	<.001	1.73 (1.06-2.98)	.03
Extramural venous invasion	1.97 (1.26-3.06)	.003	NA	NA	2.19 (1.50-3.58)	.002
Local peritoneal infiltration	NA	NA	3.63 (1.89-7.01)	<.001	NA	NA

Abbreviations: NA, not applicable; TDV, tumor deposits and extramural venous invasion.

^a^ T3 defined as greater than 5-mm depth of spread beyond the muscularis propria.

On multivariate analysis, N stage was not associated with recurrence, and only tumor deposits (HR, 1.90; 95% CI, 1.21-2.98; *P* = .006) and EMVI (HR, 1.97; 95% CI, 1.26-3.06; *P* = .003) on CT were significantly associated with overall recurrence (Table 2). Tumor deposits (HR, 2.84; 95% CI, 1.62-5.01; *P* < .001) and local peritoneal infiltration (HR, 3.63; 95% CI, 1.89-7.01; *P* < .001) on CT were significantly associated with local recurrence. Tumor deposits (HR, 1.73; 95% CI, 1.06-2.98; *P* = .03) and EMVI (HR, 2.19; 95% CI, 1.50-3.58; *P* = .002) on CT were significantly associated with distant recurrence. Lymph node disease on CT was not associated with overall, local, or distant recurrence on multivariate analysis.

**Figure 1.**
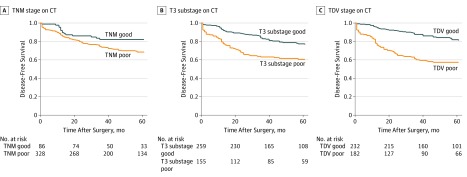
Kaplan-Meier Curves for Tumor Recurrence Using Different Staging Systems CT indicates computed tomography; TDV, tumor deposits and extramural venous invasion.

In comparisons of disease-free survival among CT staging systems to determine good and poor prognosis groups, CT-TNM did not have a significant difference between groups (HR, 1.55; 95% CI, 0.94-2.55), but CT-T3 substage classification identified poor prognosis groups (HR, 1.88; 95% CI, 1.32-2.68) (Figure 1). However, the CT–tumor deposits and EMVI classification system based on CT-detected tumor deposits and EMVI best identified a poor prognosis group (HR, 2.81; 95% CI, 1.95-4.05) (Figure 1).

**Figure 2.**
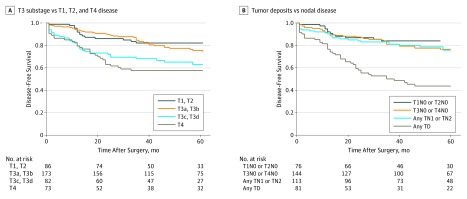
Kaplan-Meier Curves for Recurrence on Subclassification of T and N Stage on Computed Tomography T3 substage indicates T3a-T3b vs T3c-T3d category; TD, tumor deposit.

Further investigation confirmed that CT-TNM failed to achieve prognostic significance because CT-T3a and CT-T3b tumors were not associated with worse disease-free survival than CT-T1 or CT-T2 tumors when classified separately (HR, 1.04; 95% CI, 0.87-1.25) (Figure 2). Similarly, CT-T4 tumors were not associated with worse disease-free survival than CT-T3c or CT-T3d tumors (HR, 1.09; 95% CI, 0.85-1.40) (Figure 2). The nodal staging on CT was not prognostic after patients with tumor deposits on CT were separated (Figure 2).

## Discussion

The TNM stage as defined in pathologic studies has been applied for CT staging of colon cancer. Our data suggest that the TNM category does not translate to CT staging and does not provide prognostically accurate information before surgery. In contrast to the TNM system, CT-T3 substage enabled a poor prognosis group to be identified. On multivariate analysis, CT evaluation of lymph nodes was not of prognostic relevance. Computed tomography identified EMVI and tumor deposits in 41% and 20% of patients, respectively. Both EMVI and tumor deposits were independent prognostic factors for disease-free survival and therefore provided a superior preoperative staging system.

Although CT scans are routinely performed for patients with colon cancer, tumor stage has not been relevant to preoperative decision-making. With the advent of selective preoperative treatment for patients at risk of cancer recurrence, identification of a poor prognosis group through a better CT staging system is now necessary to match the improvements seen in the treatment of rectal cancer.^1,2,3^ Advanced T3 stage of colonic cancer on histopathologic analysis has been shown to be an adverse prognostic factor,^31^ but tumor stage-for-stage matching on CT imaging and pathologic specimens correlated poorly on meta-analysis.^12,13^ However, early vs advanced T3 tumor stage can be distinguished on CT with better sensitivity (87%-90%) and specificity (69%-78%)^16,19^ and acceptable interreporter reliability.^20,21^ As with pathologic results,^31^ T3 substaging on CT is prognostic for recurrence.^16,19,22^ Inclusion of all T3 sigmoid colon tumors into a poor prognosis group failed as a staging system because it did not stratify patients at risk of disease recurrence. This was explained by the equivalent recurrence rates of CT-staged T3a-T3b and T1-T2 tumors, which has not been shown previously to our knowledge. This result may explain the recent results of the FOXTROT study^17^; preoperative treatment for patients with cT3a-cT3b–staged disease would expose these patients to toxic effects for negligible benefit because their risk of recurrence is already low.

For colon cancer, T3 substage of more than 5-mm extramural spread should therefore be included on all CT reports. Given its high risk for recurrence, T3 substage poor prognosis disease should be investigated as an indication for neoadjuvant therapy.

Specialist gastrointestinal radiologists identified other variables with greater prognostic weight for disease recurrence, particularly EMVI and tumor deposits, whereas T3 substage poor prognosis disease appeared to be a surrogate measure for adverse disease features. Accurate identification of these markers individually may enable a more precise, personalized risk score for each patient. Extramural vascular invasion is a prognostic variable that is currently not included in TNM but is well known to be associated with disease recurrence,^22,32,33^ particularly for distant metastases to the liver.^34^ Tumor deposits may have previously been classified as lymph nodes on radiologic and histopathologic findings. They have now been recognized as distinct from lymph nodes by TNM,^35^ with a worse prognosis.^24,25^ Tumor deposits are associated with EMVI and are likely to represent metastases in transit after EMVI. Previous studies have shown that EMVI can be recognized on preoperative CT.^19,22,23^ In this study, tumor deposits could be differentiated from lymph nodes on CT (Figure 2B) and were associated with a significantly poorer prognosis on multivariate analysis.

### CT–T3 Substage and CT–Tumor Deposits and EMVI: Step-by-Step Guide

Identification of features of advanced colonic disease on CT requires multiplanar reconstruction on CT multidetector scanners. As shown in this study, diagnostic accuracy can be maintained across a range of different CT manufacturers using local abdomen-pelvis protocols.

#### Local Tumor Stage

**Figure 3.**
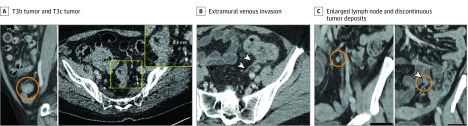
T Stage, N Stage, and Extramural Venous Invasion on Computed Tomography A, T3b tumor (within circle) shows 1- to 5-mm extramural spread; T3c tumor (inset) shows >5- to 15-mm extramural spread. B, Extramural venous invasion (arrowheads) from a tumor along the length of a vein. C, Enlarged lymph node (circle in left image) vs discontinuous tumor deposits (circle and arrowhead in right image) along the course of a vein.

Tumor spread beyond the muscularis propria can be measured on multiplanar reconstruction of the tumor.^16,19,20,23^ The CT diagnosis of a stage T3 lesion is based on the presence of tumor soft tissue extending into the pericolonic fat with a broad-based bulging or nodular configuration in continuity (Figure 3A).

#### Extramural Venous Invasion

Tumor that invades veins beyond the muscularis propria can be identified on imaging.^19,23^ On CT, the draining colonic veins can be identified as tubular structures in continuity on adjacent sections on multiplanar reconstruction. Linear or serpiginous extension of the primary tumor into the pericolonic veins can be seen because the tumor infiltrates and expands the vessel, creating an irregular contour (Figure 3B). Because this can be a subtle finding in smaller veins, it is likely that only larger-vessel EMVI is detected on CT.

#### Discontinuous Tumor Deposits

Tumor deposits are deposits of cancer cells that are discontinuous with the primary tumor and not associated with a lymph node. They can be distinguished from smoothly enlarged round lymph nodes on CT because they are more likely to have an irregular contour and mixed signal density. Although both tumor deposits and lymph nodes may appear to have an irregular contour, tumor deposits interrupt the course of a vein (best seen on multiplanar reconstruction), whereas lymph nodes lie alongside a vein (Figure 3C).

### Limitations

This study has limitations. It is possible that other centers may not be able to reproduce the level of accuracy in detection of adverse features such as advanced T3c substage, EMVI, or tumor deposits. Gastrointestinal radiologists can identify CT features that are not routinely reported.^19,20,21,22^ The prognostic importance of these features may make the case for greater specialist reporting of colon cancer. Although workshop training can improve identification of high-risk colonic tumors on CT,^8^ regular interface between specialist radiologists with the colorectal multidisciplinary team may be more important to improve an understanding of the disease and its outcomes.

Other limitations of this retrospective study were the lack of follow-up data for patients returning to their homes abroad or elsewhere in the United Kingdom and who could not be followed up. Omission of relevant covariates (eg, comorbidity) that were unavailable may have led to bias. There may also be undiscovered predictor data variables (eg, tumor biomarkers) that were confounders.

Further research should be aimed at establishing the interreader reliability of the radiologist for adverse CT prognostic features. One option is to test our T3 substage and tumor deposits and EMVI classifications against TNM in other test cohorts (eg, using CT for patients with colon cancer in the FOXTROT study,^17^ which is currently underway). This would also establish whether the adverse CT features for sigmoid colon cancer in this study carry the same prognostic weights for other sites of colon cancer.

## Conclusions

In this study, advanced T3 substage disease in colon cancer was identified on CT and was associated with a significantly adverse prognosis for disease-free survival. Tumor deposits and EMVI were independent adverse prognostic features visible on CT that were most strongly associated with worse disease-free survival. Lymph node assessment of colon cancer on CT should not be relied on since it had no prognostic value.

## Notes

Supplement. **eFigure.** Study Flowchart
