# Supplementary material for: Assessment of a Staging System for Sigmoid Colon Cancer Based on Tumor Deposits and Extramural Venous Invasion on Computed Tomography
Source: JAMA Netw Open. 2019 Dec 6;2(12):e1916987. doi: 10.1001/jamanetworkopen.2019.16987 (PMC6902773; doi:10.1001/jamanetworkopen.2019.16987)
Supplement: Supplement. — eFigure. Study Flowchart [file jamanetwopen-2-e1916987-s001.pdf]

Supplementary Online Content

D'Souza N, Shaw A, Lord A, et al. Assessment of a staging system for sigmoid colon cancer based on tumor deposits and extramural venous invasion on computed tomography. *JAMA Netw Open*. 2019;2(12):e1916987. doi:10.1001/jamanetworkopen.2019.16987

eFigure. Study Flowchart

This supplementary material has been provided by the authors to give readers additional information about their work.

© 2019 D'Souza N et al. *JAMA Network Open.*

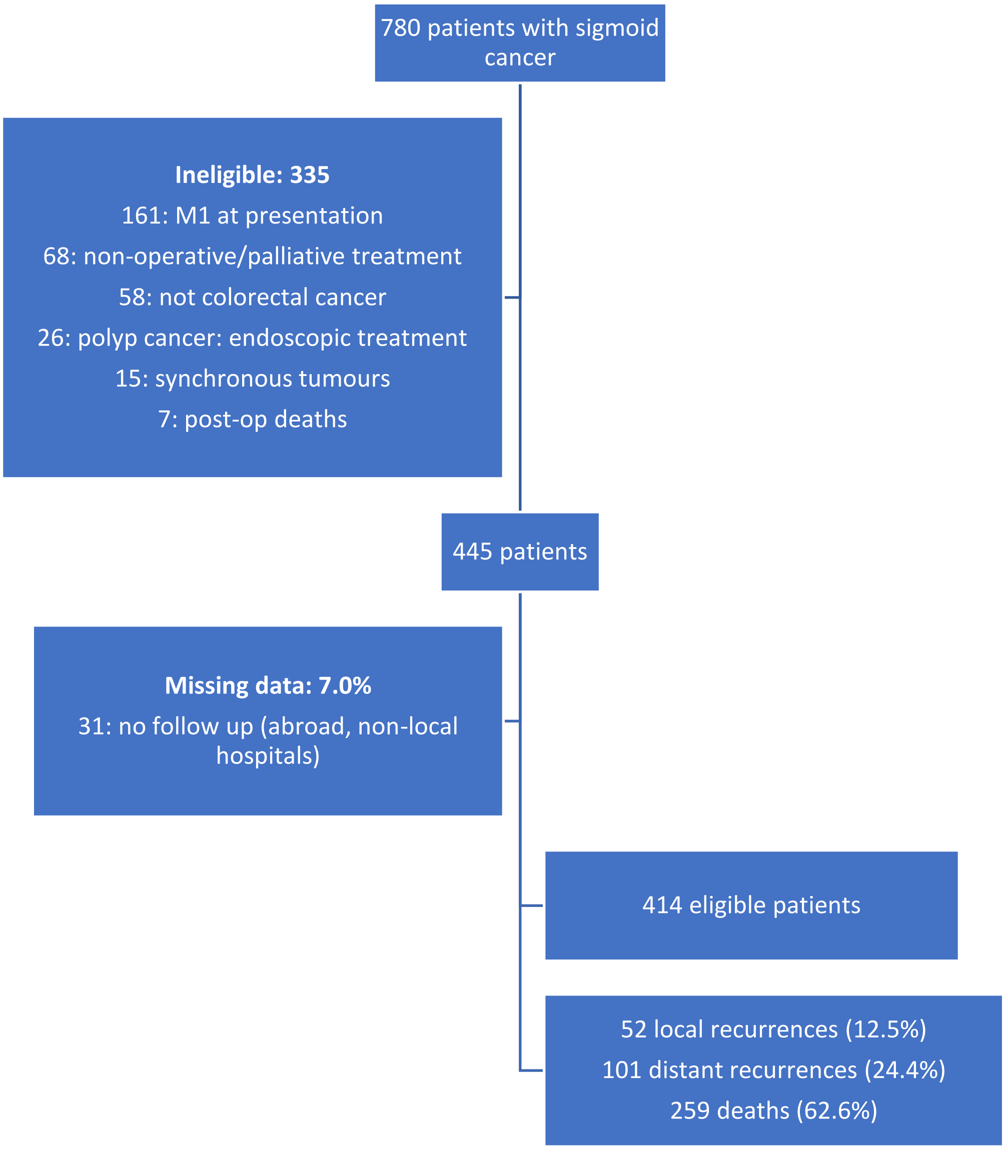

eFigure. Study Flowchart

© 2019 D'Souza N et al. *JAMA Network Open.*
